# Guttapercha Improves In Vitro Bioactivity and Dentin Remineralization Ability of a Bioglass Containing Polydimethylsiloxane-Based Root Canal Sealer

**DOI:** 10.3390/molecules28207088

**Published:** 2023-10-14

**Authors:** Paola Taddei, Michele Di Foggia, Fausto Zamparini, Carlo Prati, Maria Giovanna Gandolfi

**Affiliations:** 1 Biochemistry Unit, Department of Biomedical and Neuromotor Sciences, University of Bologna, Via Irnerio 48, 40126 Bologna, Italy; paola.taddei@unibo.it; 2 Endodontic Clinical Section, Unit of Odontostomatological Sciences, Department of Biomedical and Neuromotor Sciences, University of Bologna, Via San Vitale 59, 40136 Bologna, Italy; fausto.zamparini2@unibo.it (F.Z.); carlo.prati@unibo.it (C.P.); 3 Laboratory of Biomaterials and Oral Pathology, Unit of Odontostomatological Sciences, Department of Biomedical and Neuromotor Sciences, University of Bologna, Via San Vitale 59, 40136 Bologna, Italy; mgiovanna.gandolfi@unibo.it

**Keywords:** polydimethylsiloxane, guttapercha, trans-1,4-polyisoprene, bioglass, apatite, dentin remineralization, vibrational IR and Raman spectroscopy

## Abstract

Guttapercha (GP, trans-1,4-polyisoprene) is the most used tooth root filling material, and it must be used with an appropriate cement (typically a polydimethylsiloxane (PDMS)-based sealer) to ensure an adequate canal obturation. This study aimed to assess the bioactivity and dentin remineralization ability of a bioglass containing PDMS commercial endodontic sealer, BG-PDMS (GuttaFlow Bioseal), and to evaluate the possible influence of a GP cone (Roeko GP point) on the mineralization process. To this end, BG-PDMS disks were aged alone or in the presence of a GP cone in Hank’s Balanced Salt Solution (28 d, 37 °C). Dentin remineralization experiments were carried out under the same conditions. Micro-Raman and IR analyses demonstrated that BG-PDMS is bioactive, thanks to the formation of a silica-rich layer with nucleation sites for B-type carbonated apatite deposition. This phase was thicker when BG-PDMS was aged in the presence of GP. The two materials influenced each other because GP, which alone did not show any bioactivity, nucleated a calcium phosphate phase under these conditions. Analogously, dentin remineralization experiments showed that BG-PDMS is able to remineralize dentin, especially in the presence of GP. Under the experimental conditions, GP acted as a templating agent for calcium phosphate deposition.

## 1. Introduction

Apical periodontitis is an inflammatory lesion of the periapical bone tissue caused by bacterial proliferation into the root canal. Therefore, chemo-mechanical root canal cleaning must be followed by complete and stable sealing with filling materials to prevent re-infection. Guttapercha (GP), i.e., trans-1,4-polyisoprene, represents the material more universally approved for tooth root canal filling. Natural GP is obtained from the coagulation of latex produced by trees of the family *Sapotaceae*, mainly from *Palaquium gutta* bail [1]. The trans isomer crystallizes more easily than the cis isomer (i.e., natural rubber from *Hevea brasiliensis*), so the former is harder, more brittle, and less elastic.

Trans-1,4-polyisoprene can exist in two crystalline forms (namely, α and β), which differ in the “molecular repeat distance’’ (i.e., the distance between two consecutive -CH_3_ groups placed on the same side with the C=C double bond) [1,2]. The α and β phases are inter-convertible. When β-GP is heated, it transforms into the α-form between 42 and 49 °C and then (between 53 and 59 °C) into the amorphous phase. If α-GP is cooled at a rate higher than 0.5 °C/h, it transforms into the β-form [2]. The two forms have different properties: upon heating, α-GP shows high flowability and low viscosity and acquires adhesion properties, while β-GP has lower flowability and higher viscosity.

Thanks to its properties, guttapercha (GP), i.e., trans-1,4-polyisoprene, mainly in its β-form [3], represents the material of choice for the matrix of GP cones; it has been used in dentistry for more than 100 years to obturate root canals. The chemical composition of commercial GP cones varies from one manufacturer to the other; these devices commonly contain a matrix of trans-1,4-polyisoprene (varying between 14.5% and 21.8% in weight), waxes and resins (1–10.4% in weight, added as plasticizing agents), and zinc oxide (36.6–84.3% in weight), barium sulphate (0–31.2% in weight), together with small percentages of coloring agents and antioxidants [4,5]. Increasing the content of trans-1,4-polyisoprene in the cone seems to cause a higher brittleness; on the other hand, zinc oxide, determining a reinforcement of the matrix, improves the mechanical properties of the composite [6] and provides anti-bacterial activity [7,8]. Barium sulphate increases radiopacity.

The endodontic treatment requires chelating agents, such as EDTA or citric acid, combined with proteolytic agents, such as NaOCl. These treatments increase the risk of reduced biomechanical properties [9] and could lead to root fracture. Several long-term clinical studies also highlighted the primary importance of a correct and void-less root canal treatment [10,11]. The presence of voids between the dentin and the filling material will eventually result in a non-hermetic seal where Gram-negative anaerobic bacteria may proliferate and re-establish a root canal infection, with the need for a secondary clinical intervention [12]. The secondary reinfections lead to periapical recrudescence and a secondary intervention with lower success rates due to the technical difficulties of the treatment [10,11]. No studies have ever analyzed the chemical interactions between the two actors of the root canal obturation system—GP cones and root canal sealers—when they are placed together.

Clinically, GP cones must be used with an appropriate sealer to ensure its penetration into radicular dentine. Sealers aim to smooth any irregularities between dentin walls and the GP cone, the lateral and accessory canals, and to seal the dentinal tubules [13]. Among the materials chosen as endodontic sealers, polydimethylsiloxane (PDMS) is a silicone biocompatible polymer extensively used in biomedicine [14,15]. PDMS presents several favorable chemical and physical properties, such as a high degree of chemical inertness, thermal stability, oxidation resistance, and good mechanical properties, with a tensile strength higher than 1 MPa and ductility higher than 500%, but it exhibits relatively low bioactivity [14], i.e., low apatite-forming ability.

Bioactivity is considered crucial for relevant functional properties related to biocompatibility [16]. Bioactive materials can create an environment enhancing osteogenesis, mainly thanks to the apatite layer formed on their surface once introduced into the living body; through this layer, they can bond to living bone tissue [17,18]. Moreover, the deposition of calcium phosphates at the interface and inside the dentinal tubules improves the sealing ability of root-canal-filling materials [16]. In this context, it appears essential to design a sealing material that maintains a bond to the dentin wall and promotes dentin remineralization, thus preventing infection and dislodgement of the filling. It is generally believed that extracellular matrix proteins (mainly type I collagen, which accounts for about 90% of the organic phase of dentin) are essential in controlling apatite nucleation and growth in the dentin biomineralization process [19]. The formation of amorphous calcium phosphate (ACP) precursors has been reported in many forms of biomineralization in vitro and in vivo [20,21].

A common approach in bone and dental tissue engineering to overcome the low bioactivity of polymeric materials, such as PDMS, is to combine them with calcium phosphates (i.e., hydroxyapatite or dicalcium phosphate dihydrate), calcium silicates, or silicate glass-ceramics [14,15,22,23,24,25]. Bioglasses composed of calcium phosphate and calcium silicate (SiO_2_-CaO-Na_2_O-P_2_O_5_ bioglasses, such as 45S5 or 55S4) have been studied for their excellent bioactivity and promotion of new bone formation in vivo [26]. These stimulatory effects were attributed to released soluble silica species and Ca^2+^ ions, stimulating osteoblasts activation and proliferation [26]. As an example of a bioactive PDMS-based endodontic cement, we tested GuttaFlow Bioseal (Coltène/Whaledent AG, Altstaetten, Switzerland), a commercial bioglass containing PDMS-based root canal sealer (BG-PDMS). Compared with other sealers, its formulation shows a lower cytotoxicity on human periodontal ligament stem cells [27] and promotes their spontaneous differentiation into cementoblast-like cells [28]. In previous studies, the in vitro bioactivity of this BG-PDMS endodontic cement was demonstrated [22,23,24,29] and compared with that of a PDMS cement free from bioglass (GuttaFlow 2, Coltène/Whaledent AG, Altstaetten, Switzerland) [29].

The present study aimed to gain more insights into the in vitro apatite-forming ability of the chosen BG-PDMS cement and to test its dentin remineralization capability. As a novelty, in the present study, we tested these properties in the presence of a GP cone (Roeko GP point, Coltène/Whaledent AG, Altstaetten, Switzerland), i.e., under conditions nearer to those clinically available. For this purpose, IR and Raman spectroscopies were used to characterize the materials on a molecular scale and to monitor their bioactivity and dentin remineralization ability and the structural modifications occurring at the interface after ageing in a simulated body fluid. In particular, we wondered whether the presence of the GP cone would influence the mineralization process promoted by the BG-PDMS cement. For this purpose, it is interesting to recall that some studies demonstrated that GP (i.e., a hydrophobic material different from the hydrogels commonly used in tissue engineering) can be effective in enabling the differentiation of dental pulp stem cells and in inducing biomineralized deposits of calcium phosphates [30,31].

Moreover, bioactivity was also described for natural rubber latex from *Hevea brasiliensis*, which has been claimed as a templating agent for hydroxyapatite [32,33,34]. On the basis of the potential apatite-forming ability of GP reported in the literature, we decided to assess this property by replicating in vitro the endodontic practice of filling the root canal with a sealer (the GP cone) bound to dentin using a bioactive cement (the BG-PDMS cement). To investigate the mineralization process, we used the IR and micro-Raman fingerprint region, which has been widely used to characterize calcium phosphates in teeth and bones [35,36,37,38].

## 2. Results

### 2.1. Bioactivity Tests

**Figure 1:**
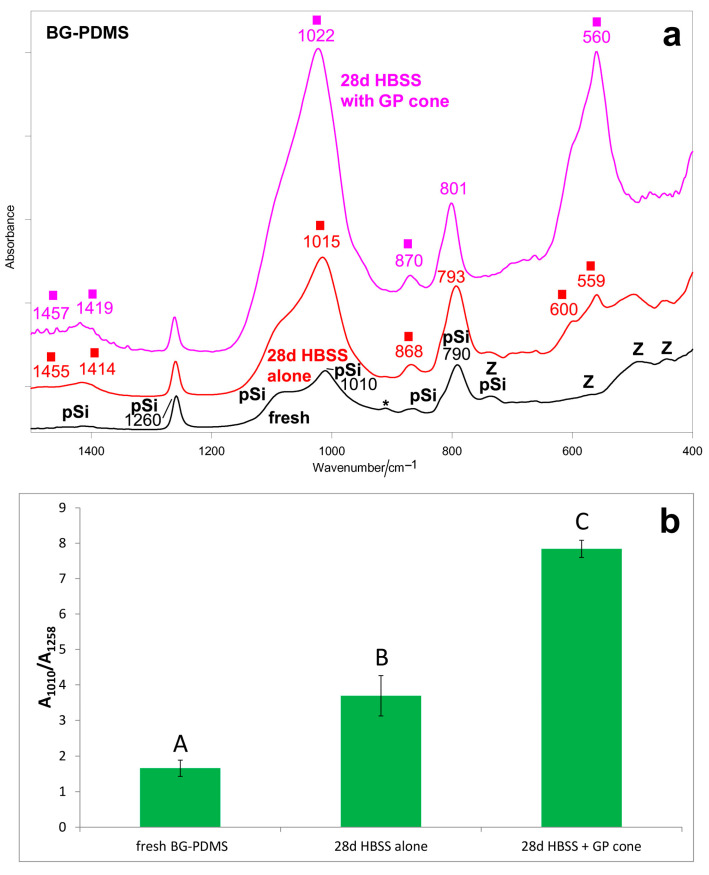
(**a**) Average IR spectra recorded on the surface of the BG-PDMS commercial cement (GuttaFlow Bioseal) before (i.e., fresh) and after ageing in HBSS for 28 days alone and in the presence of a commercial GP cone (Roeko), which was maintained in contact with the sealer. The bands assignable to polydimethylsiloxane (pSi) and monoclinic zirconia (Z) are indicated together with those specifically assigned to unreacted Si-H bonds (*) and B-type carbonated apatite (■). (**b**) IR A_1010_/A_1258_ absorbance ratio (average ± standard deviation), as calculated from the IR spectra reported in part (**a**). Different letters represent statistically significant differences between values (n = 3, *p* < 0.0001).

**Figure 2:**
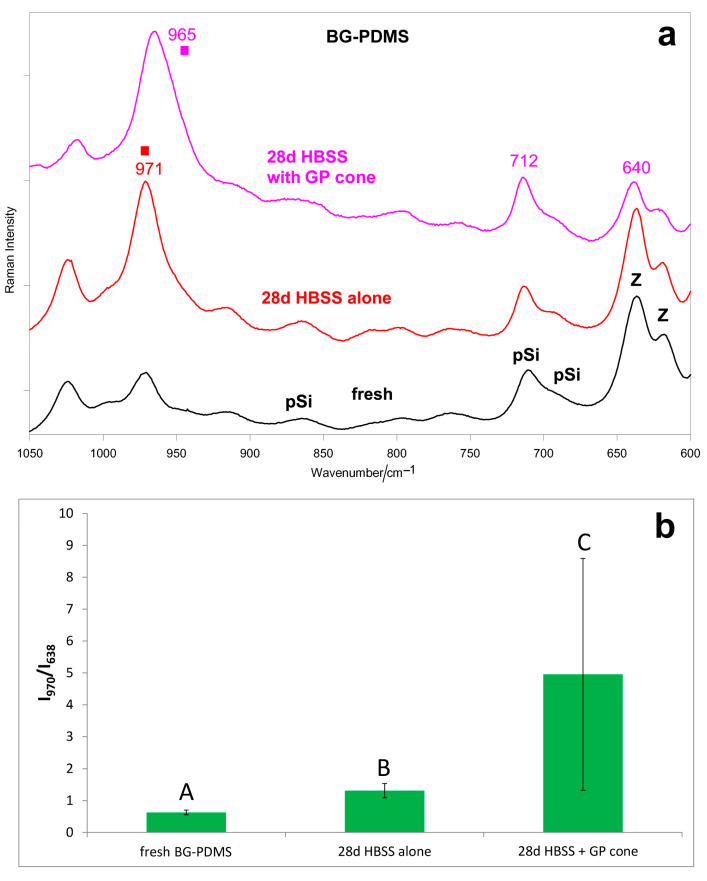
(**a**) Average micro-Raman spectra recorded on the surface of the BG-PDMS commercial cement (GuttaFlow Bioseal) before (i.e., fresh) and after ageing in HBSS for 28 days alone and in the presence of a commercial GP cone (Roeko), which was maintained in contact with the sealer. The bands assignable to polydimethylsiloxane (pSi), monoclinic zirconia (Z), and apatite (■) are indicated. (**b**) Raman I_970_/I_638_ intensity ratio (average ± standard deviation) as calculated from the micro-Raman spectra of BG-PDMS reported in part (**a**). Different letters represent statistically significant differences between values (n = 5, *p* = 0.0287).

Figure 1a and Figure 2a show the average IR and micro-Raman spectra recorded on the surface of the BG-PDMS cement (GuttaFlow Bioseal) before (i.e., fresh) and after ageing in HBSS for 28 days alone and in the presence of a GP cone (Roeko), which was maintained in contact with the sealer. In the spectrum of the fresh sample, band assignments to PDMS, and unreacted Si-H groups, monoclinic zirconia have been given according to the literature [39,40,41,42]. Bioactive glass, zinc oxide, and GP were detected in the single components of the cement [29].

The IR spectrum of the BG-PDMS sample aged alone (Figure 1a) showed distinct bands at 1455–1414 cm^−1^ (ν_3_ CO_3_^2–^ antisymmetric stretching), 1015 cm^−1^ (ν_3_ PO_4_^3–^ antisymmetric stretching), 868 cm^−1^ (ν_2_ CO_3_^2–^ out-of-plane bending), and 600–559 cm^−1^ (ν_4_ PO_4_^3–^ out-of-plane bending), all assignable to a B-type carbonated apatite [43]. The IR A_1010_/A_1258_ absorbance ratio (Figure 1b), which was identified as a marker of calcium phosphate deposition, increased accordingly (*p* < 0.05). Upon ageing, the PDMS band observed in the fresh cement at 790 cm^−1^ increased its relative intensity and shifted to 793 cm^−1^ due to the superposition of a band at about 800 cm^−1^, assignable to a silica-rich layer [44].

In the presence of the GP cone, the BG-PDMS cement was found to form a carbonated apatite deposit (Figure 1a) thicker than when aged alone, as revealed by the higher relative intensity of the bands of this phase, as well as by the value of the IR A_1010_/A_1258_ absorbance ratio (Figure 1b). However, the deposit appeared more amorphous because the phosphate bending mode was detected as a single broad band at 560 cm^−1^ [45]. The PDMS band at 790 cm^−1^ further shifted to 801 cm^−1^ and significantly strengthened due to the contribution of silica.

The micro-Raman spectra confirmed these trends (Figure 2a). When aged alone, the BG-PDMS cement formed a calcium phosphate deposit on its surface, revealed through the strengthening of the 971 cm^−1^ band. The Raman I_970_/I_638_ intensity ratio (Figure 2b), chosen as the calcium phosphate deposition spectroscopic marker, increased accordingly (*p* < 0.05). When in the presence of the GP cone, a stronger and broader apatite band at 965 cm^−1^ was detected on the surface of the BG-PDMS cement; the trend of the Raman I_965_/I_638_ intensity ratio confirms that the thickness of the deposit was higher than when the sealer was aged alone (Figure 2b). The high standard deviation associated with the micro-Raman intensity ratios obtained after ageing in HBSS can be related to the small detection area (the laser beam focused on the sample has a diameter of 1 μm) and a higher penetration depth compared to the IR/ATR technique, and it was previously reported for similar materials [29].

**Figure 3:**
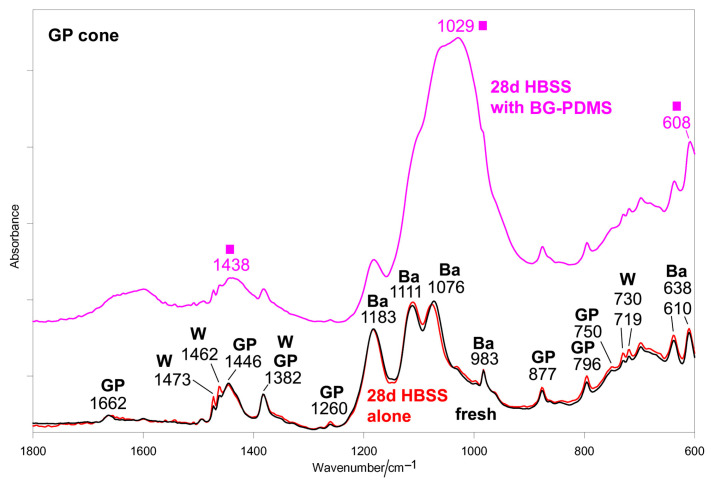
Average IR spectra recorded on the surface of the commercial GP cone (Roeko) before (i.e., fresh) and after ageing in HBSS for 28 days alone and in the presence of BG-PDMS (GuttaFlow Bioseal), which was maintained in contact with it. The bands assignable to β-guttapercha (GP), barium sulfate (Ba), wax (W), and carbonated apatite (■) are indicated.

Figure 3 shows the average IR spectra recorded on the surface of the GP cone before (i.e., fresh) and after ageing in HBSS for 28 days alone and in the presence of BG-PDMS, which was maintained in contact with it.

The spectrum of the fresh cone shows the bands of trans-1,4-polyisoprene in its β-form [46,47,48,49], barium sulfate [50], and paraffin wax, assigned according to the literature [51]. As can be easily seen, when aged alone, the GP cone underwent no significant change. On the contrary, when aged in the presence of BG-PDMS, the GP cone formed on its surface a carbonate-containing calcium phosphate deposit, as revealed by the strong band at 1029 cm^−1^ and the strengthening of the components at about 1440 and 608 cm^−1^.

Unfortunately, no information was obtained by micro-Raman spectroscopy on the GP cone due to its high fluorescence.

**Figure 4:**
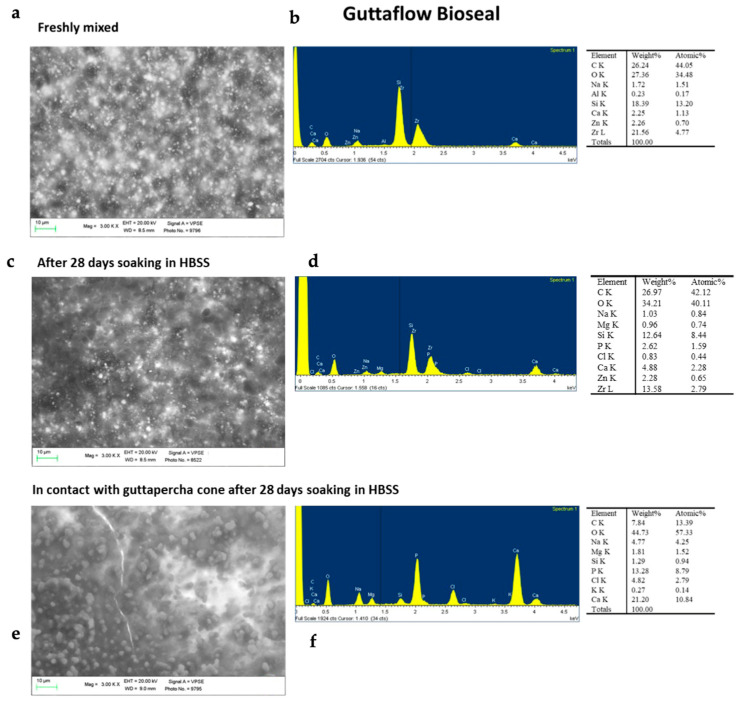
ESEM-EDX analyses of the BG-PDMS cement (GuttaFlow Bioseal) before (i.e., freshly mixed) and after ageing in HBSS for 28 days alone and in the presence of a GP cone (Roeko), which was maintained in contact with the sealer. (**a**) ESEM image at 3000X magnification revealed a homogeneous layer with few irregularities. (**b**) Compositional elements detected by EDX are assignable to the PDMS matrix (C, Si, O), bioglass (Ca, Al, Na and Si), and radiopacifier/additives (Zr, Zn). (**c**) ESEM images of the sealer disk immersed in HBSS alone revealed a surface with some globular deposits. These deposits were not uniformly distributed. (**d**) The EDX spectrum showed a lower contribution of Si and Zr, a higher contribution of Ca, and the appearance of P, suggesting the formation of a mineral layer on the surface. (**e**) ESEM image at 3000X magnification revealing a markedly different surface characterized by the presence of many granular structures and some irregularities (micro crack). (**f**) EDX revealed the appearance of P peaks, a marked increase in Ca, and a marked decrease in Si and Zr, suggesting the formation of a calcium phosphate layer that covered the sealer surface.

Figure 4 shows the ESEM-EDX analyses of the BG-PDMS cement (GuttaFlow Bioseal) before (i.e., fresh) and after ageing in HBSS for 28 days alone and in the presence of a GP cone (Roeko). The surface of the freshly mixed set sealer appears homogenous and with no irregularities (Figure 4a). Many electron-dense granules widely distributed on the whole surface were detected. EDX analysis revealed the compositional elements attributable to the PDMS matrix (silicon, carbon, oxygen), bioglass (calcium, silicon, oxygen, sodium, aluminum), and radiopacifier (zirconium and oxygen from zirconia). Zinc was detected as well.

ESEM investigation on GuttaFlow Bioseal after 28 days of ageing in HBSS alone revealed some globular structures on its surface. This layer was not uniform, and the underneath sealer was still well detectable. EDX analysis reported the decrease in Si and Zr (from GuttaFlow Bioseal sealer), the increase in Ca, and the appearance of P, suggesting a mineral layer formation.

ESEM images after 28 days of immersion in HBSS and in contact with the GP cone revealed the presence of numerous globular structures on the sealer surface, which appeared less regular. EDX analyses revealed a marked decrease in silicon, the disappearance of zirconium and zinc, a marked increase in calcium, and the appearance of a high peak of phosphorus. This finding supports the surface deposition of a mineral layer (composed of calcium carbonates and carbonated calcium phosphates) and the bioactivity of the tested sealer.

**Figure 5:**
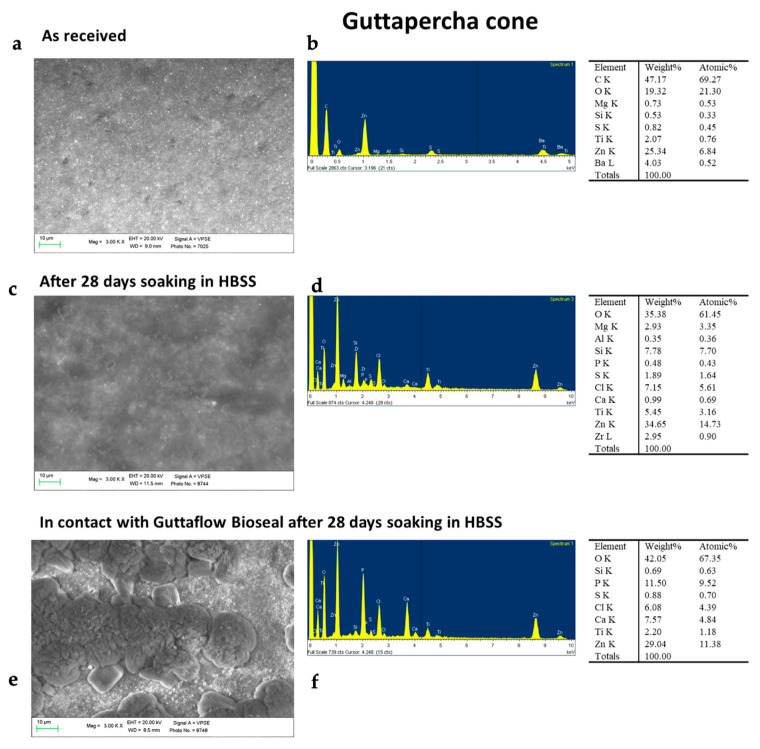
ESEM-EDX analyses of the commercial GP cone (Roeko) before (i.e., as received) and after ageing in HBSS for 28 days alone and in the presence of BG-PDMS (GuttaFlow Bioseal), which was maintained in contact with it. (**a**) ESEM image of the as-received GP cone showed a homogeneous surface. (**b**) EDX revealed the constitutional elements of GP (C), the reinforcing matrix agent (Zn and O), and the radiopacifier (Ba, S, and O). (**c**) The GP cone immersed in HBSS for 28 days alone underwent only slight modifications of its surface, as observed in the ESEM image. (**d**) The surface constitutional elements were like the as-received cone when inspected by EDX. (**e**) Differently, the GP cone placed in contact with the GuttaFlow Bioseal disk showed the formation of a mineral layer on its surface, as observed in the ESEM image. (**f**) EDX revealed strong peaks of Ca and P and a marked decrease in Si, suggesting the formation of a calcium phosphate layer.

ESEM images of the as-received GP cone revealed a homogeneous surface with no irregularities (Figure 5). The constitutional elements of the sample were revealed through EDX analysis, which detected C (assignable to trans-1,4-polyisoprene), Zn, S, O, and Ba attributable to the presence of zinc oxide (used as a matrix-strengthening agent) and barium sulphate (used as a radiopacifier). The GP cone aged for 28 days in HBSS alone showed only slight surface modifications. ESEM images revealed that the surface was not covered by any layer. EDX showed that the surface was mostly composed of C, O, Zn, S, and Si (constitutional elements of the cone), with traces of Na, K, Mg, Cl, and P (from the HBSS solution). Differently, the GP cone placed in contact with the GuttaFlow Bioseal disk and aged in HBSS for 28 days revealed a markedly different aspect. ESEM showed a mineral layer that covered the cone surface. EDX analysis revealed a marked increase in Ca and P and a marked decrease in Si, indicating the presence of a calcium phosphate layer on the cone surface.

### 2.2. Dentin Remineralization Tests

**Figure 6:**
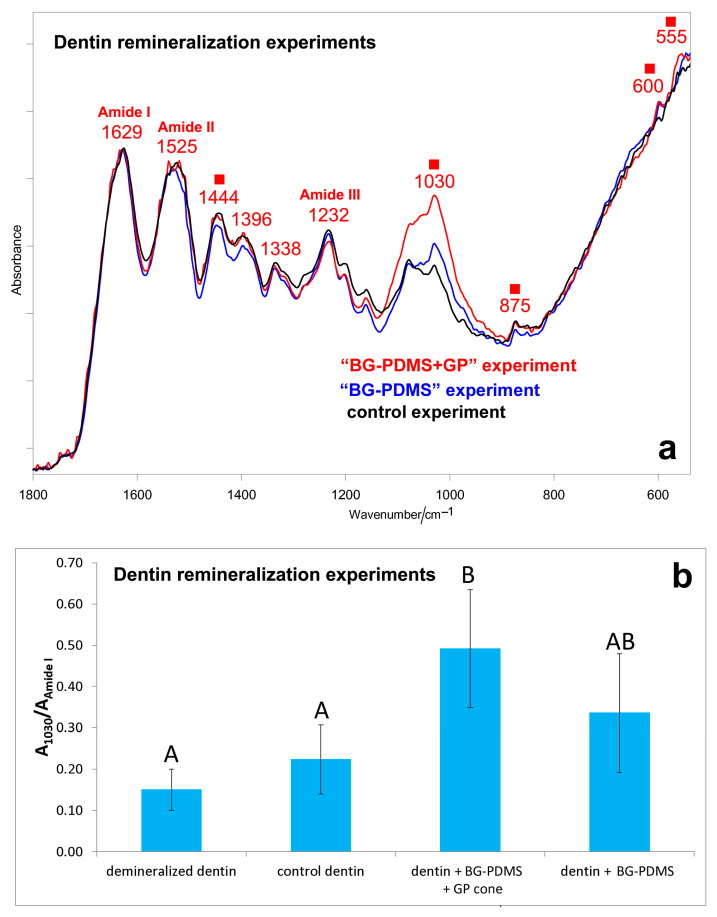
(**a**) Average IR spectra recorded on the surface of dentin slices subjected to ageing tests in HBSS for 28 days (i.e., control experiment, “BG-PDMS” experiment, “BG-PDMS+GP” experiment). The spectra are normalized to the absorbance of the Amide I band at about 1630 cm^−1^. The bands assignable to B-type carbonated apatite (■) are indicated together with Amide I, Amide II, and Amide III of collagen. (**b**) IR A_1030_/A_amide I_ absorbance ratio (average ± standard deviation) as calculated from the IR spectra of the dentin slices subjected to the above-mentioned experiments. The value corresponding to demineralized dentin is reported for comparison. Different letters represent statistically significant differences between values (n = 3, *p* = 0.0433).

Figures S1–S3, Supplementary Material, show the IR spectra of the dentin slices before (i.e., demineralized dentin) and after ageing in HBSS for 28 days in the different experiments (i.e., control, “BG-PDMS,” and “BG-PDMS+GP” experiments). Figure 6a compares the IR spectra of the dentin slices aged for 28 days in the different experiments, i.e., control (i.e., dentin alone), “BG-PDMS” (i.e., dentin maintained in contact with BG-PDMS), and “BG-PDMS+GP” (i.e., dentin maintained in contact with BG-PDMS and GP) experiments.

Upon ageing, the band at about 1030 cm^−1^ strengthened in all of the experiments (although to different extents), suggesting the deposition of a calcium phosphate phase (Figures S1–S3, Supplementary Material); moreover, the appearance of the band at about 875 cm^−1^ as well as the shift and strengthening of the band at about 1450 cm^−1^ suggest the formation of a B-type carbonated apatite. The shift and weakening of the 1406 cm^−1^ band, assignable to symmetric COO^−^ stretching [52], suggests calcium chelation by COO^−^ groups of deprotonated acidic amino acids of collagen. Amide bands of collagen, assigned according to the literature [53], underwent shifts upon remineralization, suggesting conformational rearrangements/degradation. However, these effects appeared comparable in the different experiments (Figure 6a) because the spectral profile of the Amide I, II, and III bands was nearly the same upon ageing.

**Figure 7:**
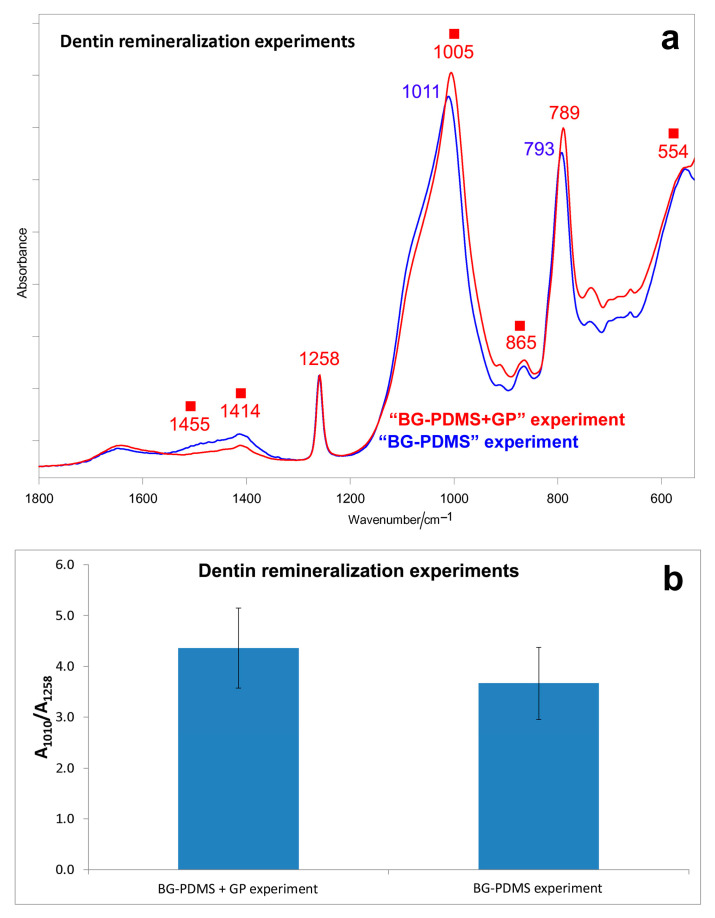
(**a**) Average IR spectra recorded on the surface of a BG-PDMS disk aged in HBSS for 28 days in dentin remineralization experiments (“BG-PDMS” and “BG-PDMS+GP” experiment). The spectra are normalized to the absorbance of the 1258 cm^−1^ band. The bands ascribable to an amorphous carbonate-containing calcium phosphate deposition are indicated with a square. (**b**) IR A_1010_/A_1258_ absorbance ratio (average ± standard deviation) as calculated from the IR spectra of the BG-PDMS disks reported above. No statistically significant differences between values were observed (n = 3, *p* = 0.6644).

As can be easily seen from the spectra reported in Figure 6a, the relative intensity of the band at about 1030 cm^−1^ increased along the series: control experiment < “BG-PDMS” experiment < “BG-PDMS+GP” experiment. The A_1030_/A_Amide I_ absorbance ratio between the strongest CaP band and Amide I of collagen was identified as a marker of dentin remineralization, and its values are reported in Figure 7b. In the presence of both BG-PDMS and GP, statistically significant differences in the A_1030_/A_Amide I_ ratio (*p* < 0.05) compared to the control were observed.

In the spectrum of the BG-PDMS disk aged in the “BG-PDMS+GP” experiment, a higher relative intensity of the 1010 cm^−1^ band was observed if compared with the “BG-PDMS” experiment (Figure 7a); however, the high standard deviation associated with the A_1010_/A_1258_ absorbance ratio (Figure 7b) made the difference not statistically significant.

**Figure 8:**
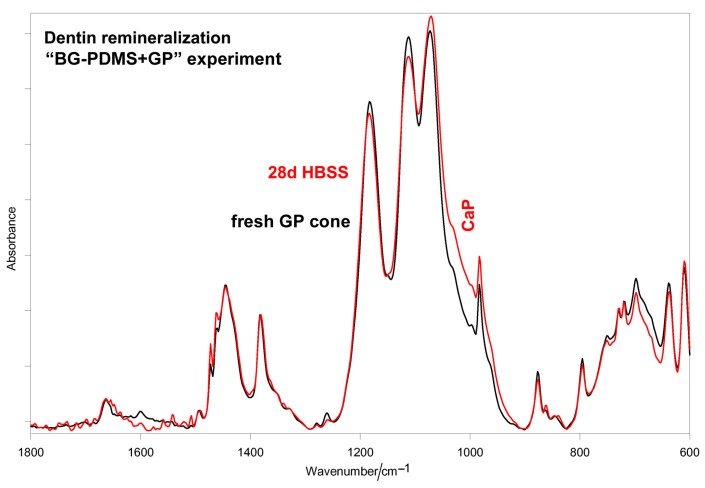
Average IR spectra recorded on the surface of the GP cone aged in HBSS for 28 days in the “Ca-PDMS+GP” dentin remineralization experiment. The spectrum of a fresh GP cone is reported for comparison. The broadening around 1000 cm^−1^ is assignable to a calcium phosphate (CaP) deposit.

In the “BG-PDMS+GP” experiment, the A_1010_/A_1258_ absorbance ratio was lower than in the analogous experiments without dentin (Figure 1b). A similar result was found for the GP cone; in the presence of dentin, a lower calcium phosphate deposition was detected on the cone (Figure 8) compared with the analogous experiment without dentin (Figure 3).

## 3. Discussion

Vibrational spectroscopic techniques were used to gain more insights into the bioactivity and dentin remineralization ability of a commercial BG-PDMS cement (i.e., GuttaFlow Bioseal) and the possible influence of GP on these properties; for this purpose, in the first part of this study, the cement and the GP cones were aged in HBSS for 28 days alone or together. Under the investigated ageing conditions, no structural changes were detected in the matrix of the samples; actually, silicone and GP-based materials were found to degrade under more drastic conditions and after ageing for longer times [54,55,56,57].

The IR A_1010_/A_1258_ absorbance ratio and the Raman I_970_/I_638_ intensity ratios (Figure 1b and Figure 2b) proved to be suitable markers to compare the samples’ bioactivity: the IR 1010 cm^−1^ band and the Raman 970 cm^−1^ band are both assigned to B-type carbonated apatite vibrations.

According to Dem et al. [58], the significant apatite-forming ability displayed by the BG-PDMS cement explains its relatively high push-out bond strength; these authors have hypothesized that their result was related to the development of a superficial layer of calcium phosphate, which can fill out the dentin voids and improve the sealing ability. The dentin remineralization tests carried out in the present study (see below for a more detailed discussion) allowed us to confirm that the BG-PDMS cement was able to remineralize dentin, especially in the presence of a GP cone (Figure 6).

On the other hand, the bioactivity displayed by BG-PDMS agrees with the long-established biochemical response elicited by 45S5 bioglass when placed in physiological fluids [59,60], i.e., the water-mediated ion exchange occurring at the glass surface. Therefore, the high apatite-forming ability of BG-PDMS indirectly confirms that the bioglass nanoparticles underwent hydration; actually, a previous study has reported that they are exposed to some extent from the hydrophobic PDMS matrix [23]. According to the literature, BG-PDMS cement possesses a relatively high percentage of water sorption [22,23], thanks to the reactions between bioglass and water. It has been reported that upon hydration of bioglass [61], the gradual dissolution of ions leads to the formation of a SiO_2_-rich layer (revealed through the band at about 800 cm^−1^, see Figure 1a) on the surface of the glass, with Si-OH groups acting as nucleation sites for calcium and phosphate ions. An amorphous layer of calcium phosphates first precipitates, which can turn to hydroxyapatite, thanks to the increased pH of the surrounding solution; then, this phase crystallizes into a biological hydroxycarbonate apatite, which is able to favor bone cells attachment and proliferation. In agreement with the proposed mechanism, the IR spectra reported in Figure 1a showed the marker bands of a B-type carbonated apatite. The bands of PDMS were still observable, suggesting that the thickness of the deposit was not yet enough to mask the cement underneath. 

To gain more insights into the bioactivity of the BG-PDMS cement, the ageing test in HBSS was repeated in the presence of a GP cone, which was maintained in contact with the sealer, i.e., under conditions more similar to those clinically available. Interestingly, the results of this experiment showed that BG-PDMS and GP influenced each other in the apatite-forming ability. As can be easily seen from the spectra reported in Figure 3, when aged alone, the GP cone did not undergo any mineralization; on the contrary, when aged in the presence of BG-PDMS, it formed on its surface a carbonate-containing apatite. At the same time, BG-PDMS formed a B-type carbonated apatite that was thicker but more amorphous than if alone (Figure 1a and Figure 2a). The latter behavior was confirmed by the trend of the ratios identified as markers of bioactivity, i.e., A_1010_/A_1258_ (IR) and I_970_/I_638_ (Raman): both ratios increased upon the ageing of BG-PDMS. However, the increase was more significant in the presence of the GP cone (Figure 1b and Figure 2b). Moreover, it must be stressed that the nature of the deposit formed on the cone was significantly different from that formed on BG-PDMS, as appears clear from the differences in the wavenumber positions of their bands (Figure 1a and Figure 3). This finding suggests that the phase formed on the cone did not originate from a physical deposition of the B-type carbonated apatite formed on the BG-PDMS material disk near it, suggesting that the underlying matrix strongly influenced the nature of the deposit.

The absence of bioactivity in the GP point is in agreement with the results reported in the literature; clinically, GP has been reported to possess an inadequate sealing ability and adhesion to root dentin [62] due to its inability to bond with it, which depends on the material’s hydrophobic nature. 

On the other hand, the bioactivity of the GP cone in the presence of BG-PDMS may be explained by the study by Al-Haddad et al. [63]. These authors succeeded in coating GP with a bioactive calcium phosphate, thanks to a harsh pretreatment with a strongly alkaline solution (5 M NaOH solution, 24 h, 60 °C), which was claimed to favor the subsequent mechanical interlocking and chemical bonding between the coating and the substrate. Similarly, Utara and Klinkaewnarong have observed that natural rubber from *Hevea brasiliensis* acted as a templating agent for hydroxyapatite deposition under basic pH conditions (pH = 11), and this process is improved by sonication [34].

The results reported in our study would suggest that mineralization occurred under significantly less drastic alkalinity conditions. Actually, it must be recalled that BG-PDMS can basify a non-buffered water medium until a pH of about 9 for a time of 28 days at least [18], while this effect is obviously less pronounced in a buffered environment [23]. On the other hand, the antimicrobial effects of bioglasses have been reported and attributed to the increase in pH of the interfacial solution to about 7.9 and above around the dissolving bioglass particles [19]. Therefore, it is not surprising that in the alkaline environment induced by BG-PDMS, the GP cone underwent mineralization, according to the mechanism proposed by Al-Haddad et al. [63]. Under the experimental conditions, GP acted as a templating agent for calcium phosphate deposition.

Based on the encouraging results obtained in this study, dentin remineralization tests were carried out to clarify the mineralizing ability of the sealers under conditions even more similar to those clinically available. Sound dentin was demineralized with EDTA, according to a consolidated protocol [64,65,66]; the absence of the B-type carbonated apatite bands in the spectra of the dentin samples treated with 17% EDTA for 2 h (Figures S1–S3, Supplementary Material) indicates that this treatment was able to demineralize dentin down to its first 2 μm of thickness, at least (i.e., the sampling depth of the ATR accessory). As previously reported [66], under experimental conditions, conformational rearrangements and changes in the hydrogen bonding pattern occurred in collagen upon demineralization. The prevailing secondary structure became β-sheet, and the content of unordered conformation increased at the expense of triple-helix, α-helix, and β-turns [66]. Despite these changes, collagen did not lose the ability to chelate calcium or promote remineralization upon ageing in HBSS, as revealed by the appearance of the bands assignable to B-type carbonated apatite (Figures S1–S3, Supplementary Material). Actually, the β-sheet conformation, prevailing in demineralized dentin, appeared particularly advantageous for mineral deposition [67,68]. The occurrence of dentin remineralization may be explained in relation to the mechanism mentioned above for the bioactivity tests. On the other hand, Efflandt et al. [69] have observed that by ageing demineralized dentin in close contact with a bioglass disk (i.e., with the two secured together with an elastic band) in artificial saliva, ions from the glass penetrated the dentin, leading to apatite formation at the interface.

Upon remineralization, conformational rearrangements occurred in dentin collagen; in particular, Amide I shifted to lower wavenumbers (Figures S1–S3, Supplementary Material), and the absorbance ratio between Amide II and Amide I (i.e., the IR A_Amide II_/A_Amide I_) ratio decreased from 0.85 to 0.67–0.68. These trends may be interpreted as a sign of calcium chelation by carbonyl groups of collagen [70] besides carboxylate (see below).

The results reported in Figure 6 showed that the presence of the GP cone together with a BG-PDMS disk improved dentin remineralization, confirming the trend obtained in in vitro bioactivity tests without dentin (Figure 1, Figure 2 and Figure 3). It must be stressed that the calcium phosphate phase formed on dentin was thinner and less mature than the B-type carbonated apatite detected in shorter-term dentin remineralization tests carried out by using calcium silicate-based cements [64,71], as expected based on the lower calcium release of the BG-PDMS commercial cement [22]. On the other hand, the lower apatite deposition observed in the present study allowed us to study dentin remineralization in the early stages of the process; the spectra reported in Figures S1–S3, Supplementary Material, showed a weakening and shift of the 1406 cm^−1^ band (rather than a strengthening, due to the contribution of the carbonate stretching mode [64,71]). This result shows that in the early stages of apatite deposition, the COO^−^ groups of deprotonated acidic amino acids play a significant role in calcium chelation and remineralization.

The calcium phosphate phase formed on the BG-PDMS disk and the GP cone in the dentin remineralization experiments was thinner and less mature (i.e., contained a lower amount of carbonate) than the phase formed on the same materials in the absence of dentin (compare Figure 1 versus Figure 7; Figure 3 versus Figure 8); for the sake of clarity, the spectra of interest are reported in Figure S4, Supplementary Material. In a previous study, we demonstrated that collagen acts as a spatial constraint to crystal deposition, a still-debated subject [72,73,74]; actually, in that study, we found that the phase nucleated by demineralized dentin upon soaking in the presence of calcium-silicate-based cements in HBSS had meanly lower crystallinity and carbonate content than the phase formed on the corresponding material disk. This aspect appeared harder to disclose in the present study due to the lower thickness of the nucleated phases; however, some spectral features reveal a different trend. As can be seen in Figure S5, Supplementary Material, the calcium phosphate phase formed on the BG-PDMS cement appeared more unordered than that formed on dentin; actually, the ν_3_ PO_4_^3–^ antisymmetric stretching band above 1000 cm^−1^ was broader in the former than in the latter (the full width at half maximum of this band was 106 cm^−1^ versus 102 cm^−1^), and the bending mode showed a single broad and weak component at 554 cm^−1^ rather than a couple of bands at 600 and 555 cm^−1^, as in dentin. At the same time, the phase formed on dentin should contain a higher amount of carbonate and acidic phosphates, as revealed by the higher relative intensity of the bands at 1444, 1076, 1056, 1050, and 875 cm^−1^ [75]. Acidic phosphates have been identified in the early stages of the mineralization process [64], and the calcium phosphate phases containing these ions represent precursors of apatite deposition [76,77,78]. No considerations can be made for the GP cone due to the low intensity of the calcium phosphate bands (Figure S5, Supplementary Material).

It must be stressed that in “BG-PDMS” and “BG-PDMS+GP” dentin remineralization experiments, the phase formed on the BG-PDMS disks appeared to be different from the B-type carbonated apatite nucleated by dentin, suggesting also, in this case, that the latter phase did not originate from a physical deposition of the material formed on the BG-PDMS disk near it. In other words, the formed apatite phase, rather than a simple deposit, was intimately bound to the collagen matrix, which, upon this interaction, underwent conformational rearrangements, in agreement with the literature [64,66].

## 4. Materials and Methods

### 4.1. Materials

As an example of a BG-PDMS endodontic cement, we tested GuttaFlow Bioseal (Coltène/Whaledent AG, Altstatten, Switzerland). According to the manufacturer, GuttaFlow Bioseal contains guttapercha powder, PDMS, a platinum catalyst, zirconium dioxide, silver (preservative), coloring, and a bioactive glass-ceramic. Material disks were prepared using PVC molds (8.0 ± 0.1 mm diameter × 1.6 ± 0.1 mm thickness).

As an example of a GP cone, we tested Roeko guttapercha points (Coltène/Whaledent AG, Altstatten, Switzerland, size #40).

### 4.2. Bioactivity Tests

BG-PDMS disks were immediately immersed vertically in 20 mL of HBSS (Hank’s Balanced Salt Solution, Lonza Walkersville, Inc, Walkersville, MD, USA) used as simulated body fluid and stored at 37 °C for 28 days. The medium was renewed weekly [64,71].

An additional test was performed under the same conditions by also immersing in the medium a GP cone, which was maintained in contact with the sealer. As a control experiment, a bioactivity test was also performed for a GP cone alone under the same conditions.

### 4.3. Dentin Remineralization Tests

Human dentin slices (5 ± 2 mm side and 0.8 ± 0.1 mm thick) from molar teeth extracted for orthodontic/surgical reasons (ethical committee approval no. 342-2019-OSS-AUSLBO-19,066 Azienda Unità Sanitaria Locale di Bologna, Local Health Unit of Bologna) were prepared and demineralized in 15 mL of EDTA 17% for 2 h at room temperature. EDTA was chosen as a demineralizing agent because it is usually used in endodontic therapy to enlarge root canals, remove the smear layer, and prepare the dentinal walls for better adhesion of the filling materials [65].

Disks of BG-PDMS cement were prepared as previously described. Paste-to-paste sealer was dispensed, mixed using a 1:1 ratio, and then compacted in molds (1.0 mm diameter × 1.0 mm height). The setting time was obtained at 37 °C and 99% RH. Set sealers were then demolded and used for the dentin remineralization tests, carried out in 20 mL of HBSS at 37 °C for 28 days. The medium was renewed weekly. In the first experiment, a BG-PDMS material disk was maintained in HBSS in contact with a demineralized dentin slice (“BG-PDMS” experiment). An additional test (“BG-PDMS+GP” experiment) was performed under the same conditions by also immersing in the medium a GP cone, which was maintained in contact with the sealer and the dentin disk. As a control experiment, a demineralized dentin slice was soaked alone (i.e., without any cement/cone) for 28 days in HBSS. After the experiments, each dentin slice was rinsed with deionized water before IR analyses.

### 4.4. Raman and IR Analyses

The surface of fresh samples and after ageing in HBSS for 28 days was analyzed using IR and Raman vibrational spectroscopies. Demineralized dentin slices used for remineralization tests were analyzed using IR spectroscopy before and after the experiments; the aged BG-PDMS cement disks and GP cones were also analyzed.

IR spectra were recorded in triplicate on a Bruker Alpha Fourier Transform FTIR spectrometer equipped with a Platinum Attenuated Total Reflectance (ATR) single reflection diamond module (penetration depth 2 μm) and a Deuterated Lanthanum α-Alanine doped TriGlycine Sulfate (DLaTGS) detector; the spectral resolution was 4 cm^−1^.

Micro-Raman spectra were obtained using an NRS-2000C Jasco spectrometer with a microscope at 100× magnification. At least five spectra were recorded on each sample and averaged. All of the spectra were recorded in backscattering conditions with 5 cm^−1^ spectral resolution using the 532 nm green diode-pumped solid-state laser (RgBLase LLC, Fremont, CA, USA) with a power of about 20 mW. A 160 K cooled digital charge-coupled device (Spec-10: 100B, Roper Scientific Inc., Sarasota, FL, USA) was used as a detector.

### 4.5. Statistical Analysis

Statistical analysis on Raman and IR data was performed with R statistical software (version 3.5.3; GNU GPL license). The data had a non-Gaussian distribution, so a non-parametric Kruskal–Wallis test was used for statistical significance. A Dunn–Bonferroni post hoc analysis was performed for any dependent variable for which the Kruskal–Wallis test was significant. All evaluation tests were two-tailed with an alpha level set to 0.05. The Kruskal–Wallis test does not compare means, but it is based on ranks and was used to verify if the rank means are different. Nevertheless, we reported the data as average values with their associated standard deviation (SD) for better readability.

### 4.6. ESEM-EDX Analyses

The surface modifications of the GP cone and BG-PDMS sealer upon ageing in HBSS were analyzed using Environmental Scanning Electron microscopy (ESEM, Zeiss EVO 50; Carl Zeiss, Oberkochen, Germany) connected to Energy Dispersive X-ray spectroscopy (EDX; Oxford INCA 350 EDS, Abingdon, UK) using computer software (Inca Energy Version 18, Abingdon, UK). The tests were performed in the same conditions as specified above; ageing was performed with the GP cone and sealer disk in contact or with both the GP cone and sealer disk alone.

All specimens were placed uncoated in the ESEM chamber under the following conditions: low vacuum (100 Pascal), accelerating voltage of 20 kV, working distance of 8.5 mm, 0.5 wt% detection level, 133 eV resolution, amplification time of 100 μs, and measuring time of 60 s for spectra. EDX analyses were made using ZAF correction [22,79,80].

## 5. Conclusions

Vibrational IR and Raman spectroscopies were successfully used to assess the bioactivity and dentin remineralization ability of a commercial BG-PDMS cement.

Ageing tests in HBSS showed that BG-PDMS was bioactive, thanks to the occurrence of hydration reactions of the bioglass particles and the formation of a silica-rich layer with nucleation sites for the deposition of a B-type carbonated apatite. This phase appeared to have a higher thickness when the material disk was aged in the presence of a GP cone. The two materials were found to influence each other because under these conditions, the cone, which alone did not show any bioactivity, nucleated a calcium phosphate phase. In other words, under experimental conditions, GP acted as a templating agent for calcium phosphate deposition, a still debated subject.

Dentin remineralization experiments showed that BG-PDMS could remineralize dentin, especially in the presence of the GP cone, confirming the findings of the tests without dentin. The results of these experiments allowed us to gain insights into the early stages of dentin remineralization, spectroscopically revealing the involvement of the COO^−^ groups of collagen in calcium chelation and apatite deposition. 

The results of this study allow us to gain more insights into relevant properties in the endodontic practice, such as the sealing ability, and show that bioactive sealers may play an innovative clinical role in endodontic therapy.

## Data Availability

Data are contained within the article or the Supplementary Material.

## Supplementary Materials

The following supporting information can be downloaded at: https://www.mdpi.com/article/10.3390/molecules28207088/s1, Figure S1: Average IR spectra recorded on the surface of a dentin slice before (i.e., demineralized dentin) and after ageing in HBSS for 28 days in the control experiment. The spectra are normalized to the Amide I band of collagen. The bands assignable to B-type carbonated apatite (■) are indicated together with the Amide I, II, and III bands of collagen; Figure S2: Average IR spectra recorded on the surface of a dentin slice before (i.e., demineralized dentin) and after ageing in HBSS for 28 days in the “BG-PDMS” experiment. The spectra are normalized to the Amide I band of collagen. The bands assignable to B-type carbonated apatite are indicated (■) together with the Amide I, II, and III bands of collagen; Figure S3: Average IR spectra recorded on the surface of a dentin slice before (i.e., demineralized dentin) and after ageing in HBSS for 28 days in the “BG-PDMS+GP” experiment. The spectra are normalized to the Amide I band of collagen. The bands assignable to B-type carbonated apatite are indicated (■) together with the Amide I, II, and III bands of collagen; Figure S4: Average IR spectra recorded on the surface of the commercial (A) BG-PDMS cement (GuttaFlow Bioseal) and (B) GP cone (Roeko) aged together in HBSS for 28 days with and without dentin. The bands assignable to the nucleated calcium phosphate phase are indicated (■); Figure S5: Average IR spectra recorded on the surface of the dentin slice, the BG-PDMS cement (GuttaFlow Bioseal), and the GP cone (Roeko) aged together in HBSS for 28 days in the “BG-PDMS+GP” experiment. The bands assignable to the nucleated calcium phosphate phase (■) are indicated (C = carbonate; H = acidic phosphate).
