# Supplementary material for: Guttapercha Improves In Vitro Bioactivity and Dentin Remineralization Ability of a Bioglass Containing Polydimethylsiloxane-Based Root Canal Sealer
Source: Molecules. 2023 Oct 14;28(20):7088. doi: 10.3390/molecules28207088 (PMC10609493; doi:10.3390/molecules28207088)
Supplement: Supplementary file 1 [file molecules-28-07088-s001.zip › molecules-2611747-supplementary.pdf]

molecules

MDPI

Supplementary Material

Guttapercha improves *in vitro* bioactivity and dentin remineralization ability of a bioglass containing polydimethylsiloxane-based root canal sealer

Paola Taddei [1], Michele Di Foggia [1,*], Fausto Zamparini [2], Carlo Prati [2] and Maria Giovanna Gandolfi [3]

[1] Biochemistry Unit, Department of Biomedical and Neuromotor Sciences, University of Bologna, Via Irnerio 48, 40126 Bologna, Italy; paola.taddei@unibo.it; michele.difoggia2@unibo.it
[2] Endodontic Clinical Section, Unit of Odontostomatological Sciences, Department of Biomedical and Neuromotor Sciences, University of Bologna, Via San Vitale 59, 40136 Bologna, Italy; fausto.zamparini2@unibo.it; carlo.prati@unibo.it
[3] Laboratory of Biomaterials and Oral Pathology, Unit of Odontostomatological Sciences, Department of Biomedical and Neuromotor Sciences, University of Bologna, Via San Vitale 59, 40136 Bologna, Italy; mgiovanna.gandolfi@unibo.it
* Correspondence: michele.difoggia2@unibo.it; Tel.: +39-051-2094281

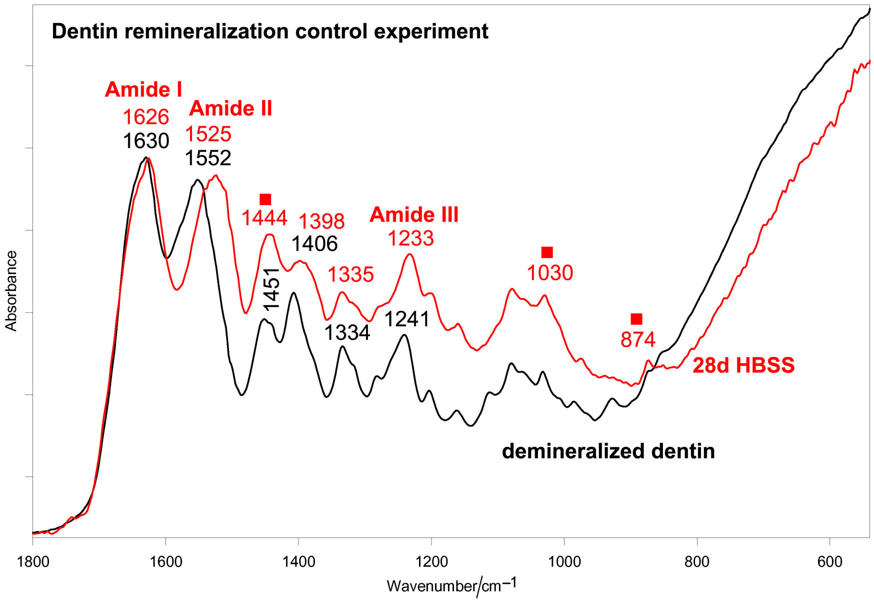

Figure S1. Average IR spectra recorded on the surface of a dentin slice before (i.e., demineralized dentin) and after ageing in HBSS for 28 days in the control experiment. The spectra are normalized to the Amide I band of collagen. The bands assignable to B-type carbonated apatite (■) are indicated together with the Amide I, II and III bands of collagen.

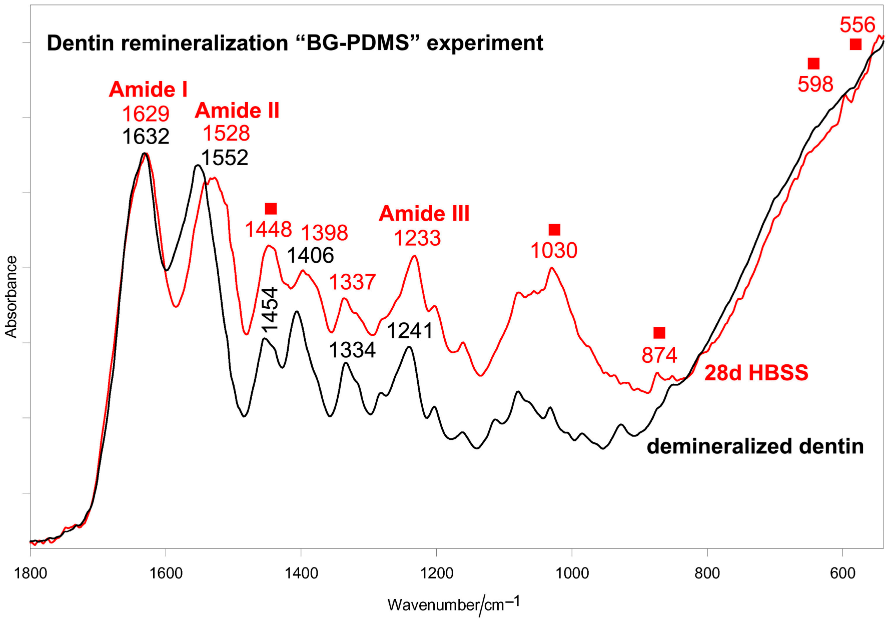

Figure S2. Average IR spectra recorded on the surface of a dentin slice before (i.e., demineralized dentin) and after ageing in HBSS for 28 days in the "BG-PDMS" experiment. The spectra are normalized to the Amide I band of collagen. The bands assignable to B-type carbonated apatite are indicated (■) together with the Amide I, II and III bands of collagen.

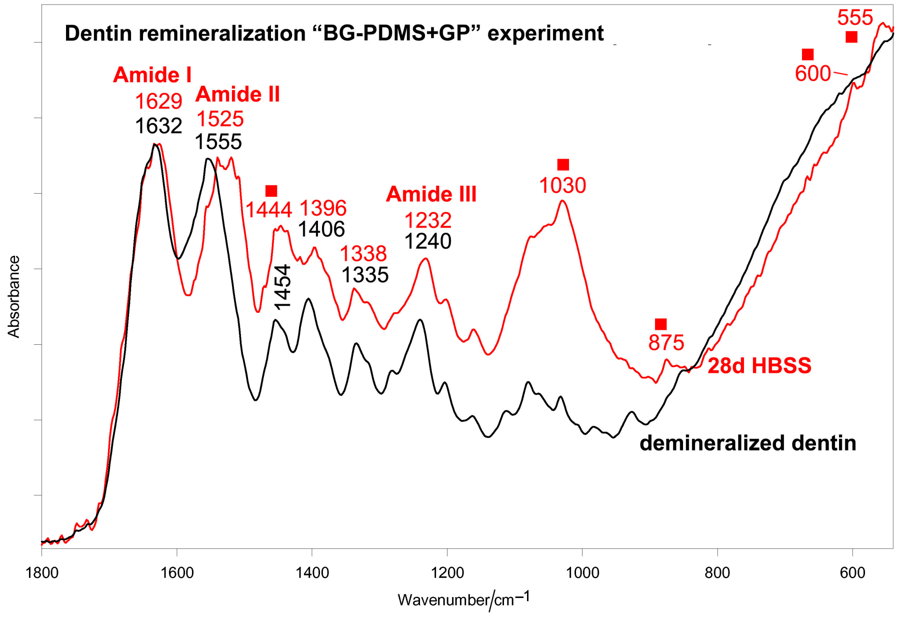

Figure S3. Average IR spectra recorded on the surface of a dentin slice before (i.e., demineralized dentin) and after ageing in HBSS for 28 days in the “BG-PDMS+GP” experiment. The spectra are normalized to the Amide I band of collagen. The bands assignable to B-type carbonated apatite are indicated (■) together with the Amide I, II and III bands of collagen.

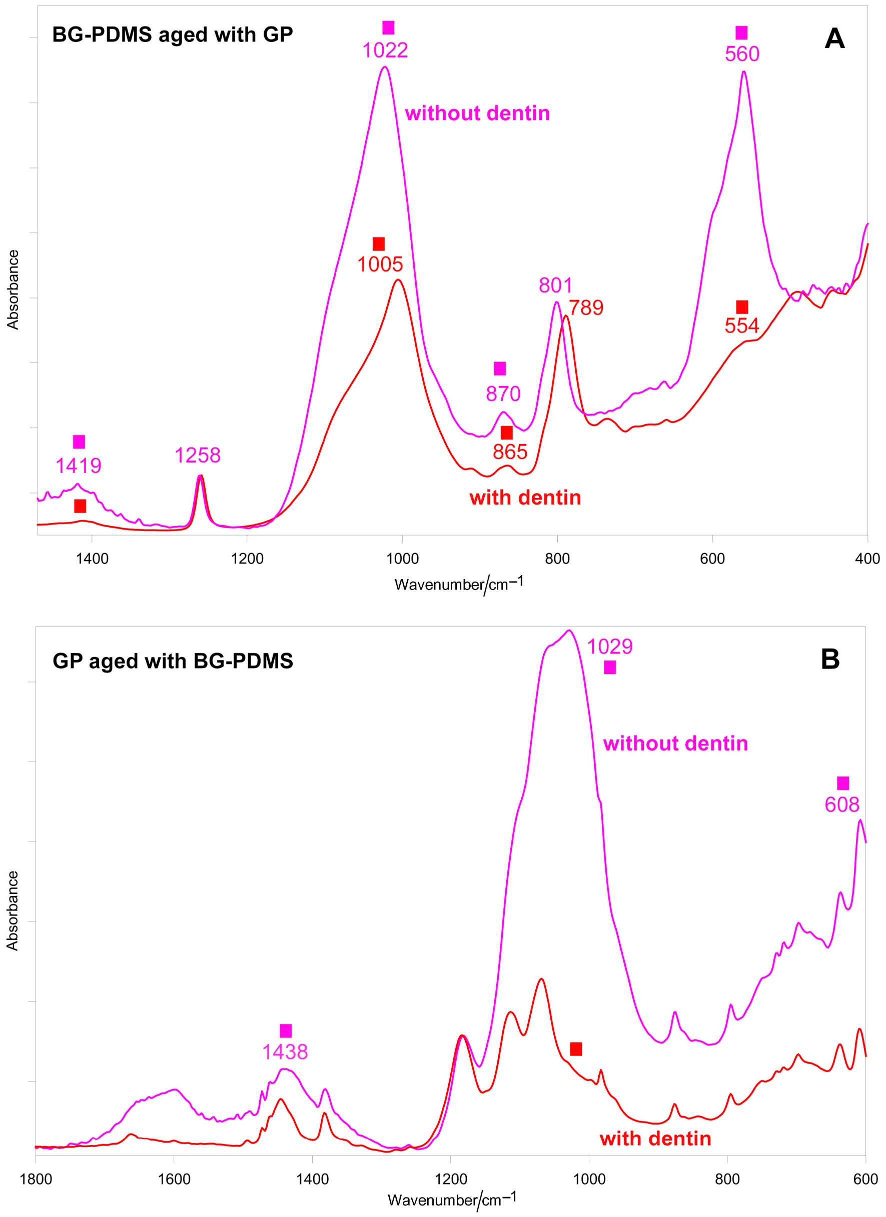

Figure S4. Average IR spectra recorded on the surface of the commercial (**A**) BG-PDMS cement (GuttaFlow Bioseal) and (**B**) GP cone (Roeko) aged together in HBSS for 28 days with and without dentin. The bands assignable to the nucleated calcium phosphate phase are indicated (■).

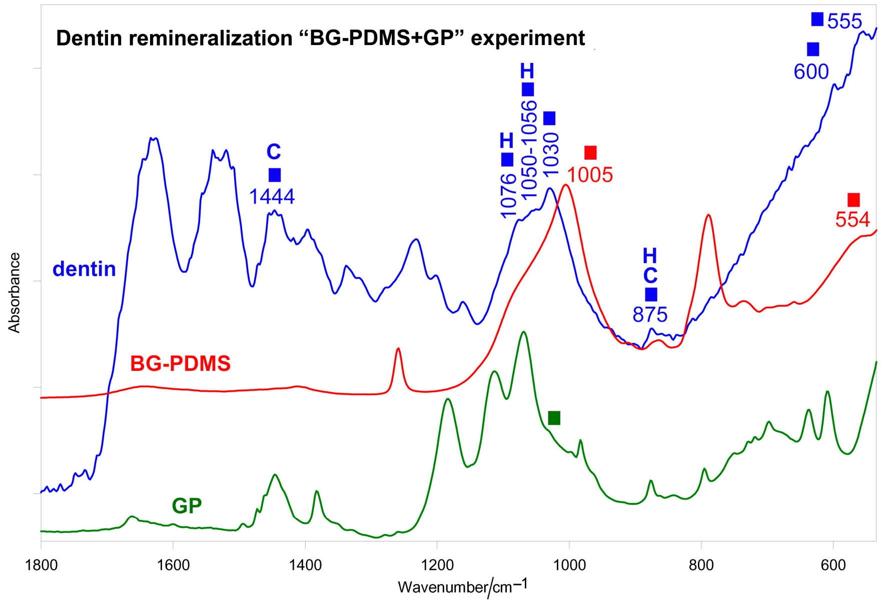

Figure S5. Average IR spectra recorded on the surface of the dentin slice, the BG-PDMS cement (GuttaFlow Bioseal) and the GP cone (Roeko) aged together in HBSS for 28 days in the "BG-PDMS+GP" experiment. The bands assignable to the nucleated calcium phosphate phase (■) are indicated (C = carbonate; H = acidic phosphate).
